# Supplementary material for: Regular Sport Activity Is Able to Reduce the Level of Genomic Damage
Source: Biology (Basel). 2023 Aug 9;12(8):1110. doi: 10.3390/biology12081110 (PMC10452097; doi:10.3390/biology12081110)
Supplement: Supplementary file 1 [file biology-12-01110-s001.zip › biology-2502780-supplementary.pdf]

Table S1. Gene Polymorphisms, Reference Sequence (rs) numbers, Primers, annealing temperatures and genotyping methodologies used

Gene Symbols	Studied polymorphisms	Polymorphism NCBI	Primer sequence	T (°C)	Methodology	PRC Product size (bp)	References
CYP1A1 exon 7	(A>G) (Ile462Val)	rs1048943	5′- AAGACCTCCCAGCGGGCAAT - 3′<br>5′- AAGACCTCCCAGCGGGCAAC - 3′<br>5′- CTCTGGTTACAGGAAGCTAT - 3′	60	ARMS-PCR	162	[40]
GSTM1	Presence/absence	rs1183423000	5- CTGGATTGTAGCAGATCATGC - 3′<br>5′- CTGCCCTACTTGATTGATGGG - 3′	65	PCR	273	[41]
GSTT1	Presence/Absence	rs1601993659	5′- TTCCTTACTGGTCCTCACATCTC - 3′<br>5′- TCACCGGATCATGGCCAGCA - 3′	63	PCR	480	[42]
XRCC1	(C>T) (Arg194Trp)	rs1799782	5′- GCCCCGTCCCAGGTA – 3′<br>5′- AGCCCCAAGACCCTTTCACT - 3′	60	RFLP (MspI)	Undigested Product: 383 bp<br>C-allele = 346 + 37 bp<br>T-allele = 383 bp	[43]
XPC exon 15	(A>C) (Lys939Gln)	rs2228001	5′ – ACCAGCTCTCAAGCAGAAGC – 3′<br>5′ – CTGCCTCAGTTTGCCTTCTC – 3′	60	RFLP (Pvu II)	Undigestyed Product: 281 bp<br>A-allele = 281<br>C-allele = 150+131 bp	[44]
